# Auditory and Speech Outcomes Following Transcutaneous Bone Conduction Hearing Implantation

**DOI:** 10.7759/cureus.74257

**Published:** 2024-11-22

**Authors:** Mohamed Garrada, Mohammed K Alnoury, Omar K AlNoury, Abdulsalam Alqutub, Omar A Alsulami, Haythem R Abuzinadah, Khaled I Alnoury

**Affiliations:** 1 Otolaryngology - Head and Neck Surgery, King Abdulaziz University Faculty of Medicine, Jeddah, SAU; 2 Medicine, King Abdulaziz University Faculty of Medicine, Jeddah, SAU; 3 Otolaryngology- Head and Neck Surgery, King Abdulaziz University Faculty of Medicine, Jeddah, SAU

**Keywords:** auditory outcome, baha, bone-anchored hearing device, bone conduction, cochlear baha attract system, hearing performance, magnetic system

## Abstract

Objectives: Implantable bone conduction hearing devices offer excellent auditory rehabilitation. Transcutaneous devices, which use an implanted magnet, are gaining popularity due to higher skin complications associated with traditional percutaneous devices. The Cochlear^TM^ Baha® Attract System (Cochlear Corporation, Sydney, Australia) is a transcutaneous device and is regarded as a passive transcutaneous implant. This study, encompassing children and adults, aims to evaluate audiological and speech outcomes, as well as postoperative complications after the implantation of the Cochlear^TM^ Baha® Attract System.

Methods: This is a retrospective cohort analysis of patients who underwent the Cochlear^TM^ Baha® Attract procedure from January 2017 to December 2020. Demographics, pre-and postoperative hearing thresholds, speech discrimination testing, hearing-aided thresholds, and complications were assessed.

Results: A total of 13 patients underwent the Cochlear^TM^ Baha® Attract surgery. The most common cause of implantation was microtia and aural atresia. Seven patients had mixed hearing loss (MHL), four had conductive hearing loss (CHL), and two patients had single-sided sensorineural deafness (SSD). A statistically significant improvement in hearing and speech understanding was observed with the device compared to preoperative unaided hearing (p-value = 0.00001). The overall mean for the gain at frequencies 0.5 to 4 kilohertz (kHz) was 31 decibels (dB). On average, speech discrimination scores improved by 36% after surgery. Postoperative wound healing and skin condition remained stable, with no major soft tissue-related complications. A patient developed a skin reaction at the implant site and was treated conservatively. All patients were satisfied and maintained device usage.

Conclusions: The Cochlear^TM^ Baha® Attract System, classified as a passive transcutaneous bone conduction hearing implant, provides excellent auditory outcomes in patients with conductive, mixed, or single-sided sensorineural hearing loss, with minimal soft tissue complications in both children and adults. More research is necessary to directly compare passive and active transcutaneous bone conduction devices.

## Introduction

Bone conduction hearing implants transmit sound directly through the skull to the inner ear [1]. These implants have been used successfully for more than three decades, offering excellent hearing outcomes for patients with conductive hearing loss (CHL), mixed hearing loss (MHL), and single-sided sensorineural deafness (SSD) [2].

Percutaneous bone conduction hearing implants have been widely used with good auditory outcomes. However, they have certain drawbacks, particularly with respect to the percutaneous abutment, which is associated with cosmesis and skin complications surrounding the abutment [3, 4]. The transcutaneous bone conduction hearing implant known as the Cochlear^TM^ Baha® Attract System implant (Cochlear Corporation, Sydney, Australia) was approved by the US Food and Drug Administration (FDA) in 2013 [5-7]. Unlike its percutaneous counterpart, this system does not rely on a skin-penetrating abutment; instead, it utilizes an osseointegrated implant attached to an internal magnet underneath the skin and coupled to an external magnet to retain the sound processor. It falls into the category of passive bone-conducting hearing implant. Research has shown that the combination of advanced sound processing, stable single-point fixation in the bone, and evenly distributed contact pressure results in efficient sound transmission and minimal skin complications [8,9].

In light of the scarcity of published data on passive transcutaneous bone conduction implants, the current study attempts to assess speech and auditory functions after passive transcutaneous bone conduction hearing implants.

## Materials and methods

Participants and methods

Study Design and Ethics Statement

This retrospective cohort study was approved by the King Abdulaziz University Research Ethics Committee (No. 437-22), which was carried out at King Abdulaziz University Hospital (KAUH) in Jeddah, Saudi Arabia.

Inclusion criteria included the following [10]: 1) Adults and children five years and older; 2) Participants with MHL and CHL should have average bone conduction threshold<45dB hearing level (HL); 3) SSD with normal bone thresholds at the unaffected ear; 4) Limited benefit from traditional hearing aids; 5) Motivation to participate in a rehabilitation program.

Exclusion criteria included: 1) Children less than five years of age; 2) Patients with conditions predisposed to an abnormal bone formation such as Paget's disease, osteoporosis, or previous radiation; 3) Patients with pathology close to the ear requiring MRI surveillance.

Preoperative Assessment

At the baseline visit before surgery, pure tone audiograms were obtained, including masked/unmasked air and bone conduction thresholds with speech recognition scores using live speech monosyllabic words. All patients were tested with a Baha® soft band to ensure patient satisfaction.

Surgical Technique

All candidates underwent implantation of the CochlearTM Baha® Attract System following the manufacturer’s standard technique [11]. The surgical technique involves making a wide C-shaped incision around the implantation site at a distance of at least 15 millimeters from the magnet edge. Soft tissue thickness is measured in three positions (anterior magnet edge, middle of magnet, posterior magnet edge). The incision and drilling steps were performed following the manufacturer's guidelines [11].

Postoperative Hearing Performance Assessment

Aided free-field hearing threshold measurement with the Baha sound processor was performed. Subjects were seated in a sound-treated room one meter away from a loudspeaker situated in the 0° azimuth. Free-field warble tone thresholds with the Baha processor using the behavioral map and volume level that would be used in speech perception testing were determined in the frequency range 250-4000 Hertz (Hz) in octave intervals. These pure tones were generated by an interacoustic audiometer (Clinical Audiometer AC40, Interacoustics A/S, Middelfart, Denmark) that had been calibrated to accepted standards (American National Standards Institute (ANSI) 1969). Thresholds were obtained in 5-dB increments using a routine clinically modified method of limits where subjects were asked to indicate hearing a sound by pressing a button, raising a hand, or by conditioned play audiometry in the case of younger children. The speech discrimination test of monosyllabic words for the subjects with the device based on the behavioral map was performed in a quiet room with minimal distractions. The patient should first understand the instructions, occasionally with the aid of visual cues, before proceeding. A list of monosyllabic words was presented to the patient to discriminate the words and repeat them. This was followed by a list of 25 words. The correct and incorrect responses received scores of one and zero, respectively. Accurate repetition of the word is considered a correct response. The percentage of correct responses is determined by recording the correct responses in relation to the total number of stimuli.

## Results

A total of 13 patients underwent the Cochlear^TM^ Baha® Attract System surgery. There were nine males and four females, whose ages ranged from five to 68 years (Table 1). As shown in Table 2, the most common cause of implantation was microtia and aural atresia. Seven patients had MHL, four had CHL, and two had SSD. 

**Table 1:** Distribution of the Demographic Data of the Studied Cases (N = 13)

Demographic Data	Number of Patients (Percentage)
Sex	
Male	9 (69.2%)
Female	4 (30.8%)
Age	
< 18 years (Pediatric)	8 (61.5%)
≥ 18 years (Adult)	5 (38.5%)
Min - Max (Years)	May-68
Mean (Years) ± SD	26.08 ± 20.79
Median (Years)	19

**Table 2:** Clinical Characteristics of Implanted Patients (N = 13)

Characteristics	Number of Patients (%)
Indications	
Microtia and Aural Atresia	6 (46.2%)
Single-Sided Deafness (SSD)	2 (15.4%)
Chronic Ear Discharge	2 (15.4%)
Post-mastoidectomy	2 (15.4%)
Syndromic Cause of Conductive Hearing Loss (Crouzon Syndrome)	1 (7.7%)
Side	
Right Ear	9 (69.2%)
Left Ear	2 (15.4%)
Bilateral	2 (15.4%)
Type of Hearing Loss	
Conductive Hearing Loss (CHL)	4 (30.8%)
Sensorineural Hearing Loss (SNHL)	2 (15.4%)
Mixed Hearing Loss (MHL)	7 (53.8%)

All surgeries were performed under general anesthesia without any intraoperative complications. The mean skin thickness measured was 6 mm, and the typical duration of surgery was 40 minutes. The Baha® processor device was fitted two weeks after surgery, and there were no incidents of implant exposure or loss. Six subjects utilized a magnet with a strength rating of two; four individuals required a magnet strength of three; three needed a magnet strength of five; and the remaining two used a magnet strength of four. During follow-up visits, one patient developed a skin reaction and erythema on the magnet site and was treated with local antibiotic therapy. All patients were satisfied with the implant.

**Figure 1:**
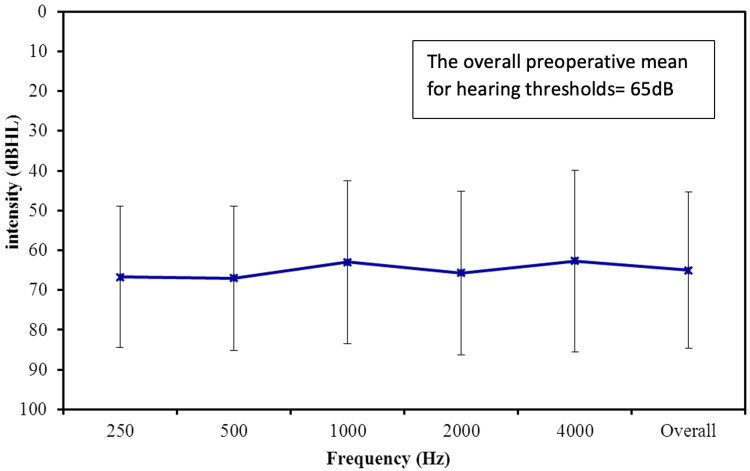
The Preoperative Mean Frequency-Specific Pure-Tone Hearing Threshold (N = 13)

**Figure 2:**
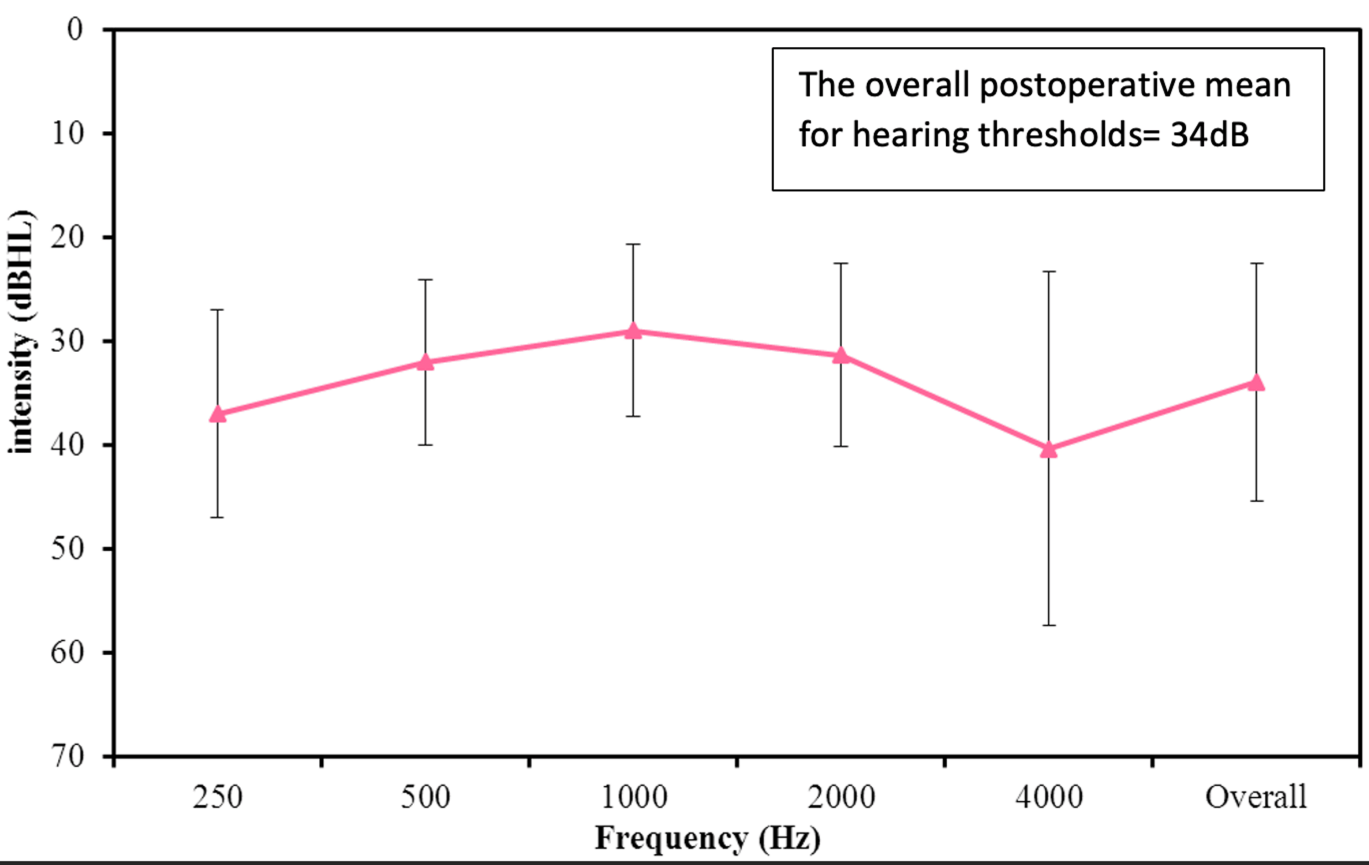
The Postoperative Mean Frequency-Specific Pure-Tone Hearing Threshold (N = 13)

**Figure 3:**
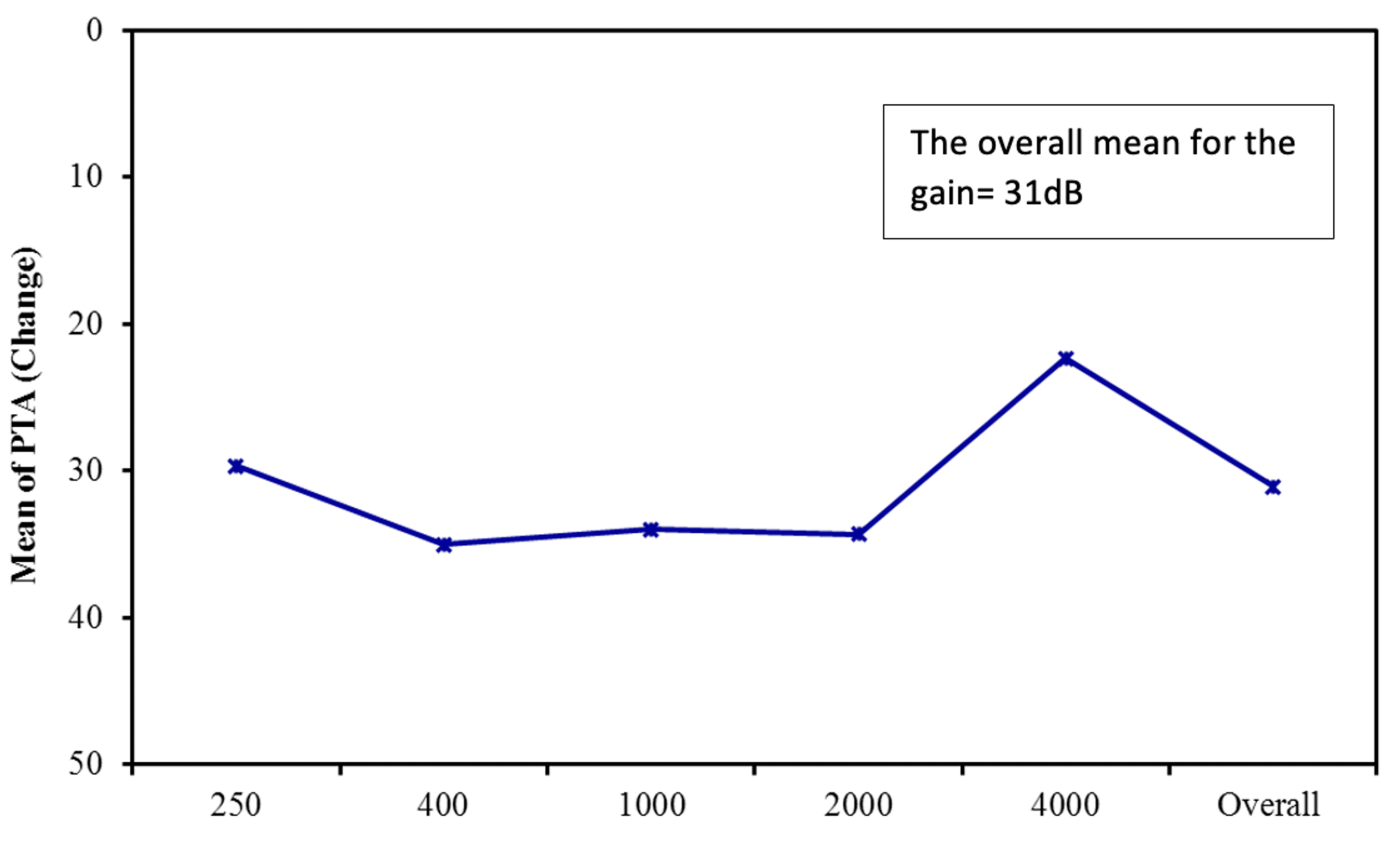
The Mean for the Frequency-Specific Pure-Tone Hearing Threshold Gain (N = 13) PTA: pure-tone average

**Figure 4:**
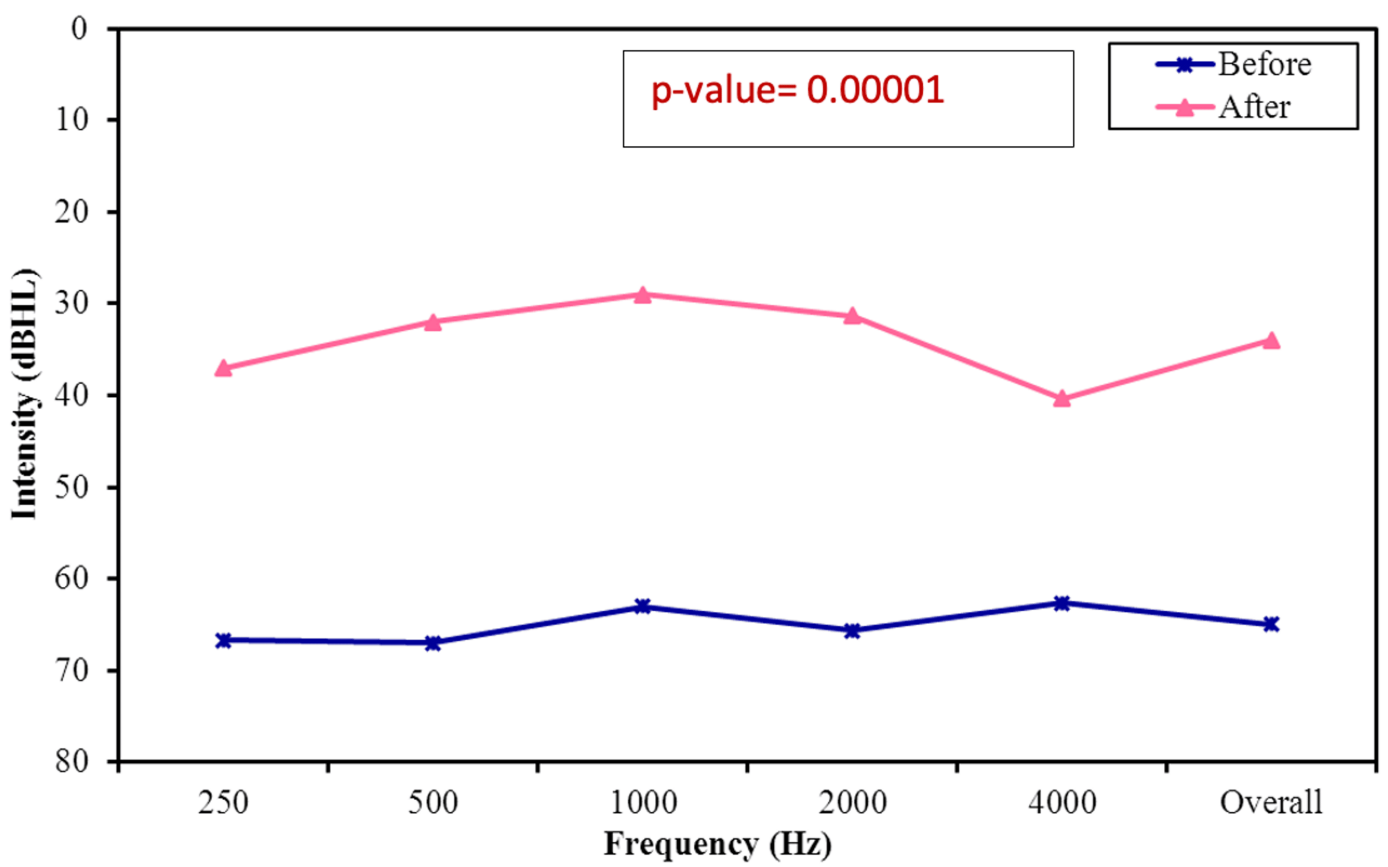
Aided Versus Unaided Mean Pure-Tone Average (PTA) Results (Mean of 500, 1000, 2000, and 4000 Hz)

**Figure 5:**
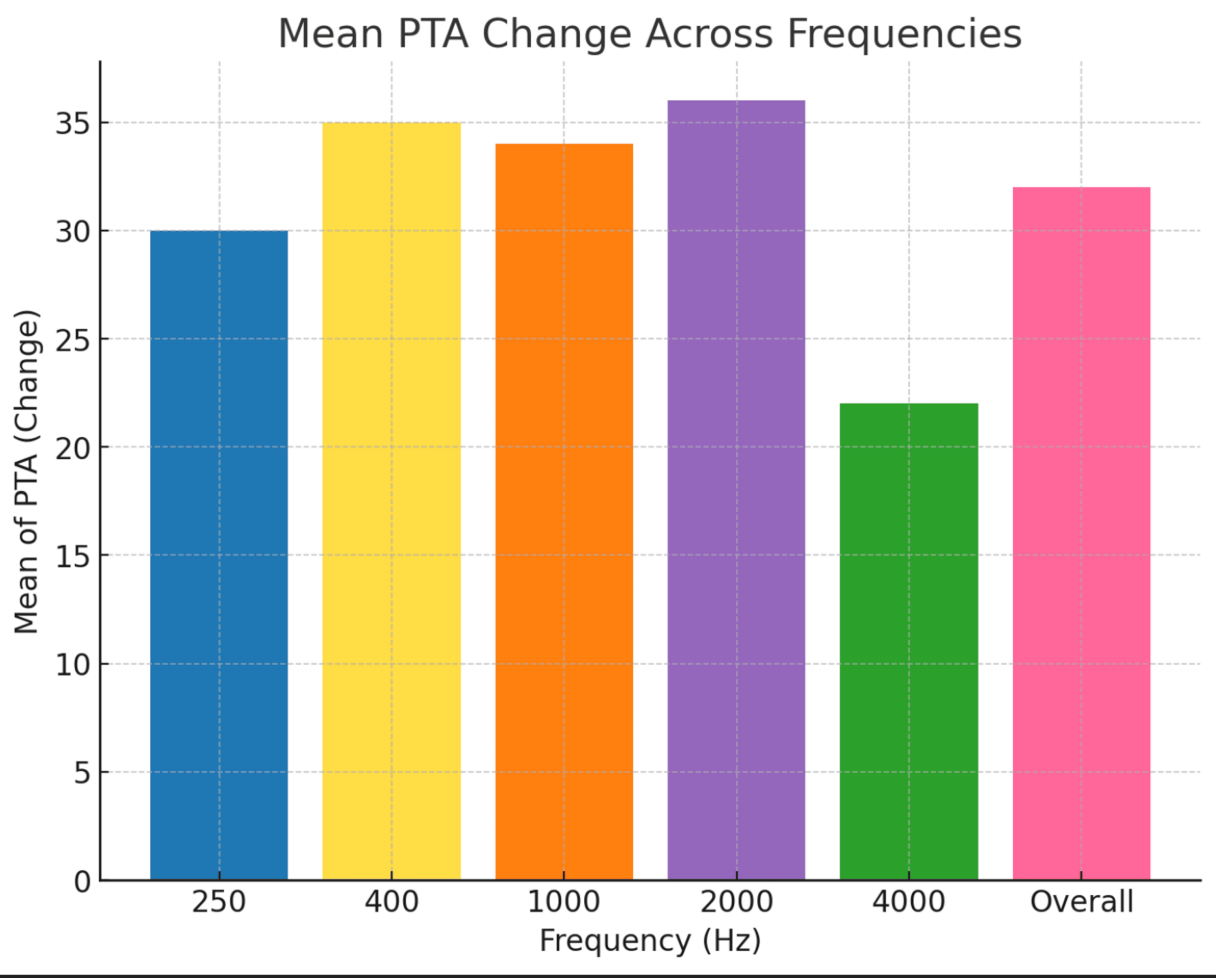
The Overall Mean Frequency-Specific Gain (N = 13) PTA: pure-tone average

**Figure 6:**
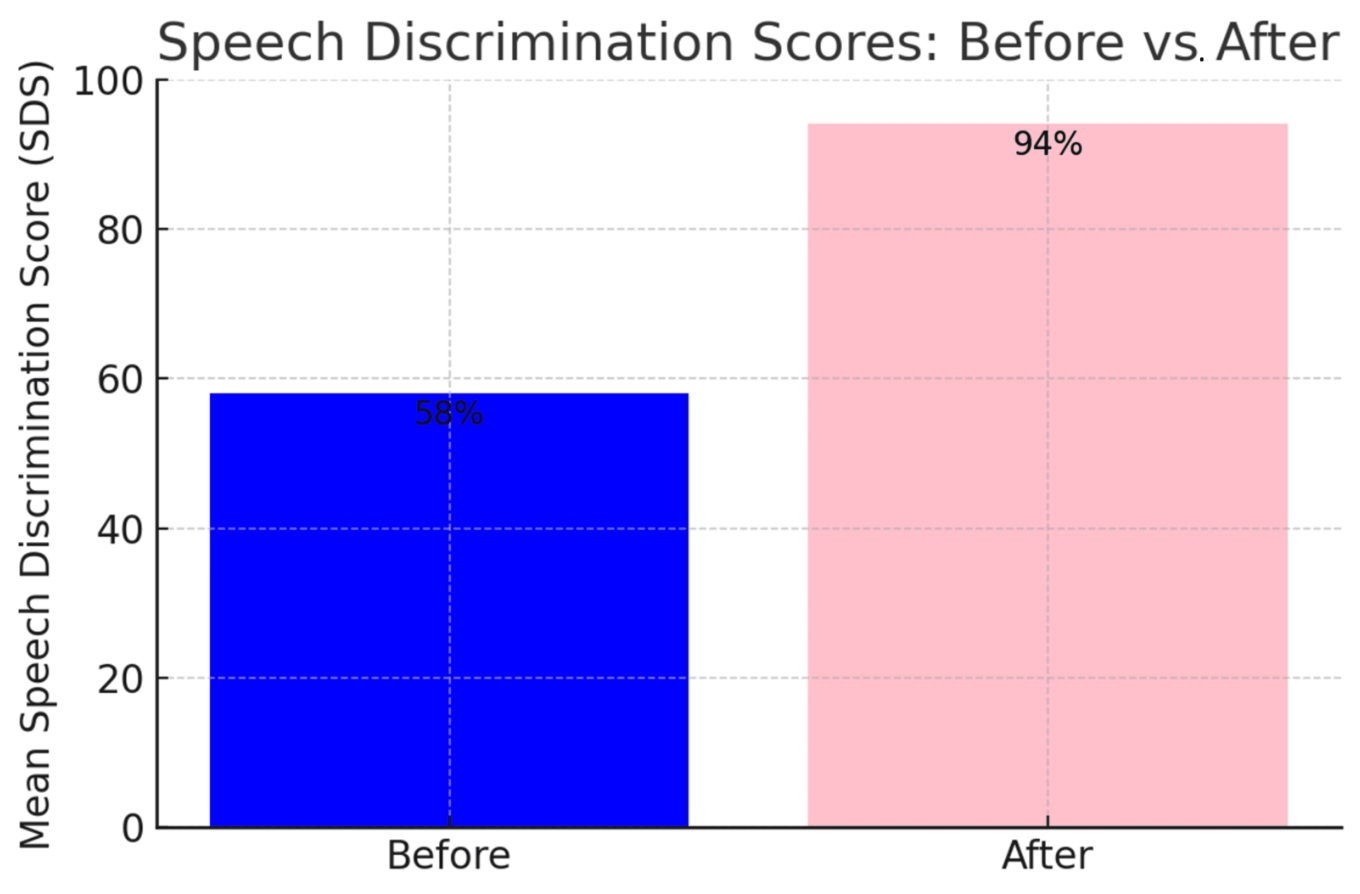
Speech Discrimination Scores Phonetically Balanced (PB) Monosyllabic Word List in Percentage Before and After CochlearTM Baha® Attract System Surgery

The average preoperative hearing thresholds for frequencies 500 to 4000 Hz were 65 dB (Figure 1). Postoperatively, the overall mean thresholds with the device were reduced to 34 dB, as shown in Figure 2. The overall mean gain in all frequencies was 31 dB (Figure 3). Using a paired samples t-test, the aided pure tone audiometry thresholds showed a statistically significant improvement in comparison to the unaided condition (p-value = 0.00001) (Figure 4). In particular, a better gain was observed for mid-frequencies (500-2000 Hz), while a smaller gain was observed for the lower (250 Hz) and higher (4000 Hz) frequencies, as shown in Figure 5. During the speech discrimination testing, a mean speech discrimination score (SDS) of 58% preoperatively compared to 94% postoperatively was found, indicating a significant difference in SDS percentage between conditions with and without the device (Table 3 and Figure 6).

**Table 3:** Speech Discrimination Scores Phonetically Balanced (PB) Monosyllabic Word List in Percentage Before and After Cochlear™ Baha Attract Surgery (N = 13)

Speech Discrimination Score (SDS)	Preoperative (%)	Postoperative (%)
Min. – Max.	12 – 80	72 – 100
Mean ± SD	58.80 ± 20.89	94 ± 8.25
Median	60	100

## Discussion

This study describes our experience in a tertiary center that describes the auditory functional outcomes of the Cochlear^TM^ Baha® Attract System in both children and adults. The Baha Attract system in our series was used in patients with CHL, MHL, and SSD who did not benefit from conventional hearing aids. Although the primary indication is for patients with CHL caused by external and middle ear pathologies, the procedure can also be considered for patients with surgical mastoid cavities or those who are facing challenges with conventional hearing aids [9, 12].

The Cochlear^TM^ Baha® Attract System offers advantages over skin-penetrating bone-anchored systems by providing improved cosmesis and eliminating the need for daily cleaning of the implant site [13]. With advancements in sound processor (SP) technology, effective sound transmission is achievable despite the inherent soft tissue attenuation associated with magnetic bone conduction hearing implants. Although the system must ensure reliable retention of SP to ensure good clinical outcomes, our study indicates that it generally does not cause irritation or discomfort to the skin, except for one patient who was treated conservatively, which is comparable to the incidence reported in the literature [14]. No major complications, such as implant loss or skin necrosis, were observed.

The present study demonstrated a statistically significant improvement in hearing performance compared to unaided hearing (p-value = 0.00001). The aided free-field hearing threshold measurements with the device suggest significant improvement at all frequencies, with the most improvement observed in the range of speech frequency, which is comparable to what has been reported in the literature [15]. At 4000 Hz, performance slowly declines as expected due to the known soft tissue attenuation effect, mainly affecting high frequencies as reported in the literature [16, 17]. It is anticipated that the high-frequency thresholds aided by the algorithm could be improved by increasing the gain at high frequencies in the programming software. Furthermore, a statistically significant improvement was observed when comparing the SDS with and without the device. (mean SDS of 58% preoperatively compared to 94% postoperatively).

The limitations of the study include small sample size and the lack of a comparison group with other forms of bone-conduction hearing implants. Furthermore, the study was carried out prior to our center's approval of the OSIA® 2 system, a new active transcutaneous implant developed by Cochlear Corporation [18]. On the other hand, this study is unique in that it is one of the few studies that report on hearing outcomes after the Cochlear^TM^ Baha® Attract System implantation and includes even younger children: the youngest participant was five years old. Furthermore, the study used a strong methodology for hearing evaluation that included pure-tone average (PTA) and speech evaluation, allowing for a detailed comparison of pre-and post-implantation hearing results.

## Conclusions

Our study demonstrates the excellent auditory outcome offered by the Cochlear^TM^ Baha® Attract System transcutaneous system for patients with CHL, MHL, or SSD. The BAHA® Attract System restores hearing through coupling without the need for percutaneous bone implant procedures. A notable benefit is the decreased risk of soft tissue complications often seen with traditional percutaneous systems. This enhanced both patient comfort and aesthetic outcomes. Further studies are required to compare it with active transcutaneous bone conduction implants.
